# Ethylene Diamine Tetra Acetate-Induced Pseudo Thrombocytopenia (EDTA-PTCP) in an Adolescent: A Case Report

**DOI:** 10.7759/cureus.38545

**Published:** 2023-05-04

**Authors:** Adarsh Vardhan Tangella, Ravindra Kumar Peta, Deepak C Yadlapalli, Digumarthi Raghunadha Rao, Manikanta Swamy M

**Affiliations:** 1 Internal Medicine, Andhra Medical College, Visakhapatnam, IND; 2 Internal Medicine, King George Hospital, Visakhapatnam, IND; 3 Medical Oncology, GSL Medical College, Rajahmundry, IND; 4 Pathology, Satya Scans and Laboratories, Rajahmundry, IND

**Keywords:** edta-pctp, ethylene diamine tetra acetate-induced pseudo thrombocytopenia (edta-ptcp), ethylene diamine tetra acetate, auto-analyser, thrombocytopenia, case report, smear examination, edta-induced pseudothrombocytopenia

## Abstract

Ethylene diamine tetra acetate-induced pseudo thrombocytopenia (EDTA-PTCP) is a fictitious laboratory condition that is associated with platelet clumping, leading to falsely low platelet counts. This fictitious occurrence can lead to expensive, time-consuming, and invasive diagnostic procedures. It may also result in the application of unnecessary therapies,^ ^although it is not linked to any hemorrhagic symptoms or platelet malfunction. This emphasizes the necessity of verifying laboratory results from automated analyzers in every patient with thrombocytopenia with a peripheral smear, particularly when they are out of proportion when compared to the clinical features. When using hematology analyzers, EDTA-induced pseudo thrombocytopenia can be missed. In cases of isolated thrombocytopenia, this can be easily avoided by performing a simple visual peripheral blood smear check, hence making it an important differential for thrombocytopenia on an automatic analyzer report, which has to be ruled out. Here, we present the case of an adolescent who presented to us with low platelet counts and was diagnosed with EDTA-PTCP after proper evaluation.

## Introduction

Ethylene diamine tetra acetate-induced pseudo thrombocytopenia (EDTA-PTCP) is a spurious laboratory phenomenon that is present in 0.1%-0.2% of the general population [1-3]. This in vitro phenomenon is characterized by platelet aggregates in EDTA-anticoagulated blood due to antiplatelet autoantibodies [4]. It may result in the application of unnecessary therapies and diagnostic procedures, such as platelet transfusions [5], bone marrow aspiration and biopsy [6], and occasionally long-term steroid therapy or even splenectomy [7-9], although it is not linked to any hemorrhagic symptoms or platelet malfunction. Several case studies where emergency treatments were withheld have been described [10]. Additionally, it could trigger an unwelcome alarm in the patient's mind, which might prompt precautionary and restrictive behaviors to stop bleeding manifestations [5]. Hence, it is essential for healthcare professionals to be aware of this phenomenon, especially when managing patients with low platelet counts.

## Case presentation

**Figure 1:**
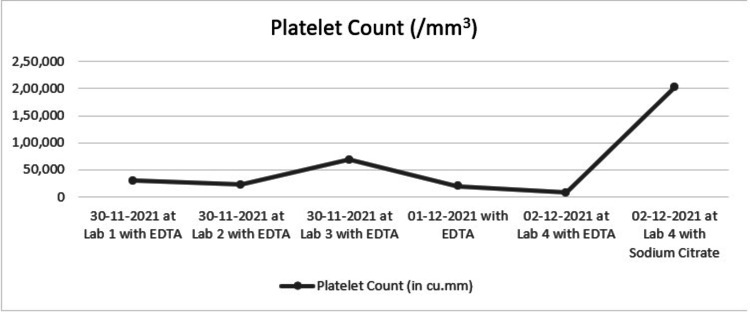
Variation of platelet counts between labs based on the type of anticoagulant used EDTA: ethylene diamine tetra acetate

A 17-year-old girl from a small-town hospital presented with a low platelet count and a recent episode of fever. The patient was healthy and had no complaints of weakness, rashes, or bleeding. Her blood counts were tested as a part of routine investigations, which revealed thrombocytopenia but no other symptoms such as bleeding or easy bruising. She was subjected to repeated laboratory testing of platelet counts in different labs to rule out possible disparities between the methods used. Figure 1 shows a summary of her platelet counts.

Tests for scrub typhus serology, rapid testing for the malarial parasite antigen, rapid testing for the non-structural protein 1 antigen (NS-1), and immunoglobulin M (IgM) and IgG antibodies for dengue were all negative. To rule out the possibility of marrow dysfunction, bone marrow aspiration and biopsy were performed, revealing a hypercellular, normoblastic bone marrow with adequate iron reserves and megakaryocytic hyperplasia. A summary of her laboratory test results is presented in Table 1.

**Table 1:** Laboratory investigations with normal reference values RBC: red blood cells; MCV: mean corpuscular volume; MCH: mean corpuscular hemoglobin; MCHC: mean corpuscular hemoglobin concentration; WBC: white blood cells; ALT: alanine transaminase; AST: aspartate transaminase; ESR: erythrocyte sedimentation rate; ALP: alkaline phosphatase; Gamma GT: gamma-glutamyl transferase; A/G ratio: albumin/globulin ratio; INR: international normalized ratio; HIV: human immunodeficiency virus; HbsAg: hepatitis B surface antigen; HCV: hepatitis C; T3: triiodothyronine; T4: thyroxine; TSH: thyroid stimulating hormone

Investigation	Results	Biological reference value
Hemoglobin	12.3 gms%	12-16 gms%
RBC count	4.57 million cells/cu.mm	4.5-5.5 million cells/cu.mm
Hematocrit	39.1%	36%-46%
MCV	85.6 fl	84-99 fl
MCH	26.9 pg	25-33 pg
MCHC	31.5 gm/dl	31-37 gm/dl
Total WBC count	3370 cells/cu.mm	4000-11000 cells/ cu.mm
Differential count: Neutrophils, lymphocytes, monocytes, eosinophils, basophils	39%, 51%, 06%, 04%, 00%	55-75%, 15-40%, 2-10%, 2-6%, 0-1%
ESR	38 mm in 1^st^ hour	0-10 mm in 1^st^ hour
Reticulocyte count	0.5%	0.5-2.5%
Serum bilirubin (T)	0.30 mg/dl	0.3-1.2 mg/dl
Serum bilirubin (direct)	0.04 mg/dl	< 0.2 mg/dl
Serum bilirubin (indirect)	0.26 mg/dl	
ALT	48 IU/L	< 35 IU/L (female adult)
AST	32 IU/L	< 35 IU/L (female adult)
ALP	63 IU/L	30-120 IU/L
Gamma GT	19 IU/L	<38 IU/L (female adult)
Total proteins	7.4 g/dl	6.6-8.3 g/dl
Albumin	4.3 g/dl	3.5-5.0 g/dl
Globulin	3.1 g/dl	3.5-5.0 g/dl
A/G ratio	1.38	
Blood glucose (random)	86 mg/dl	70-140 mg/dl
Blood urea nitrogen	6.54 mg/dl	6.5-21.0 mg/dl
Blood urea	14 mg/dl	17-43 mg/dl
Serum creatinine	0.55 mg/dl	0.4-1.2 mg/dl
Serum uric acid	2.57 mg/dl	2.6-6.0 mg/dl (female adult)
Serum phosphorous	3.27 mg/dl	2.5-5.0 mg/dl
Serum calcium	9.9 mg/dl	8.8-10.6 mg/dl
Serum sodium	140.7 mmol/L	135-155 mmol/L
Serum potassium	3.82 mmol/L	3.5-5.5 mmol/L
Serum Cchlorides	101.7 mmol/L	98-108 mmol/L
Prothrombin time	16.58 seconds	11-15 seconds
Activated partial thromboplastin time	37.4 seconds	27-34 seconds
INR	1.24	
HIV	Negative for HIV 1 and HIV2	
HbsAg	Negative	
HCV	Negative	
Direct Coomb’s test	Negative	
Indirect Coomb’s test	Negative	
T3	116.0 ng/dl	91-164 ng/dl
T4	14.58 μg/dl	5.48 14.28 μg/dl
TSH	0.823 μIU/ml	0.36-5.80 μIU/ml

**Figure 2:**
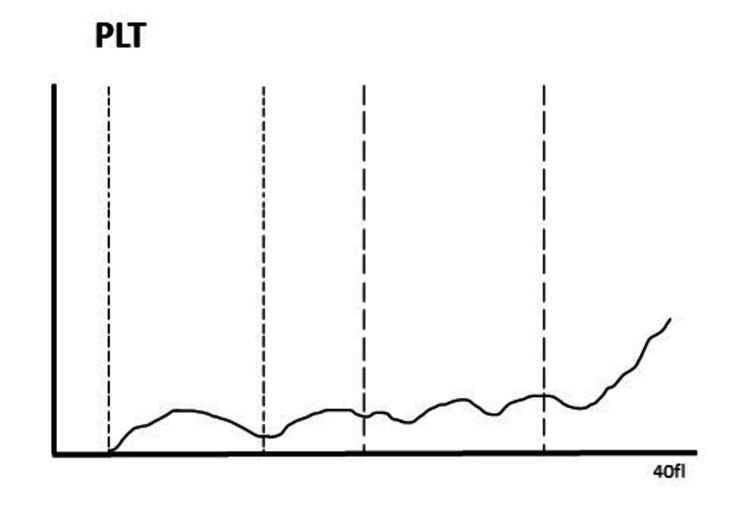
Histogram showing the EDTA sample with a platelet count of 8,000 cells/mm3 and a typical sawtooth pattern seen in EDTA-PTCP PLT: platelets; EDTA-PTCP: ethylene diamine tetra acetate-induced pseudo thrombocytopenia

Her immature platelet fraction was 39.2. The platelet count as determined using an automated analyzer and the EDTA anti-coagulated sample was 8000/mm3, and the platelet histogram exhibited a typical "sawtooth" pattern rather than a smooth curve, indicating the necessity of a peripheral smear (Figure 2).

**Figure 3:**
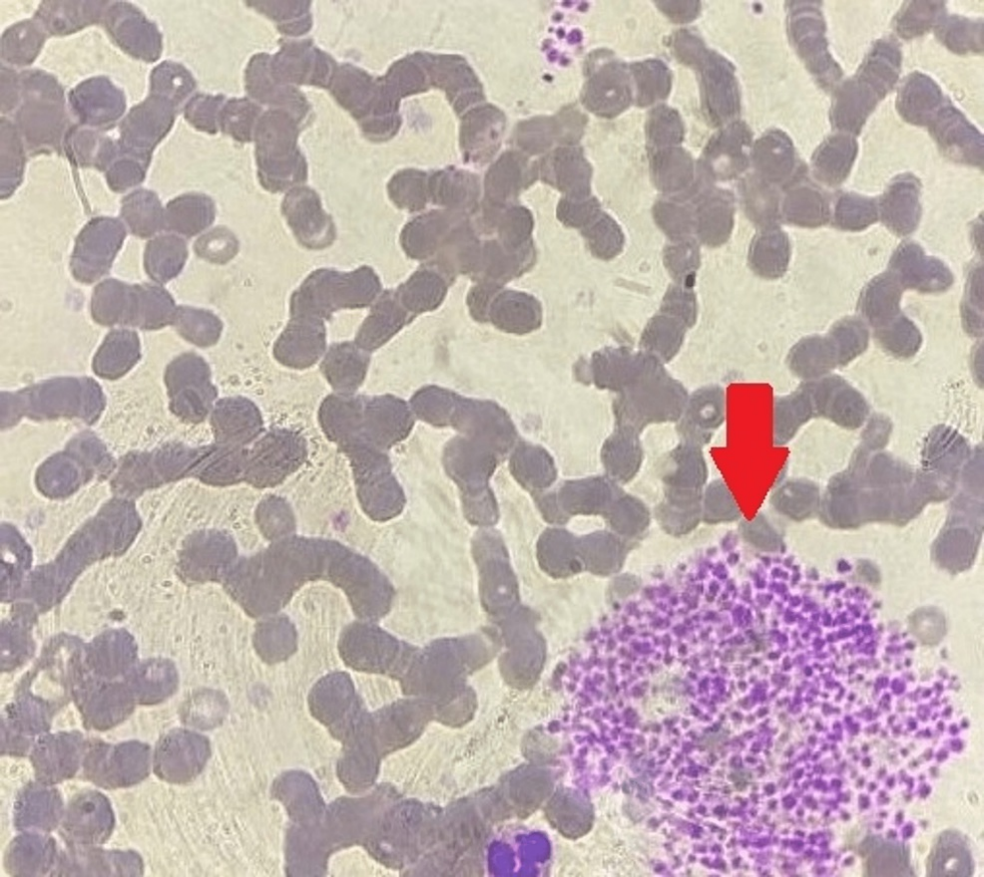
Peripheral smear performed using EDTA as an anticoagulant shows platelet clumps (red arrow) EDTA: ethylene diamine tetra acetate

**Figure 4:**
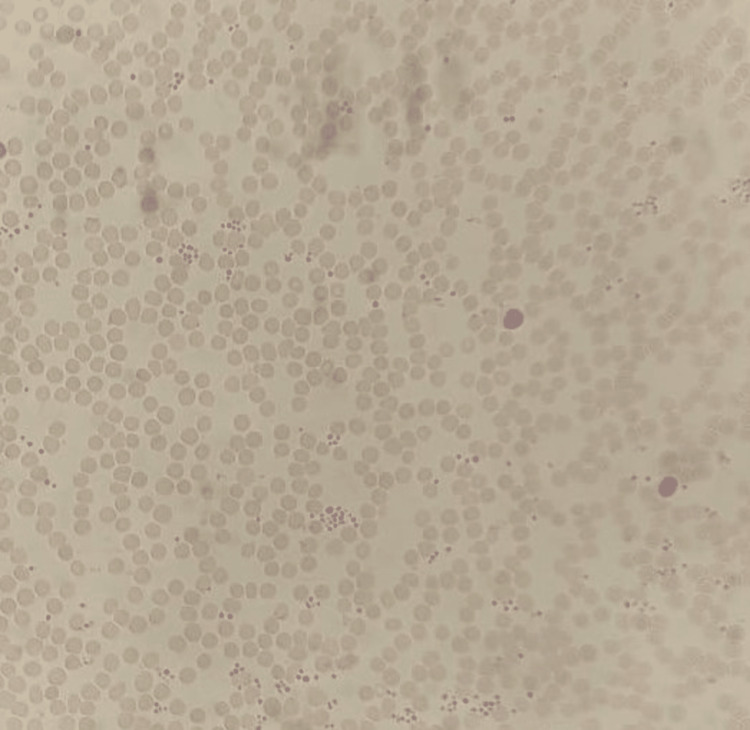
Direct peripheral smear from the sample without using any anticoagulant

When EDTA was used as the anticoagulant, a visual examination of the peripheral smear revealed normocytic and normochromic RBCs, while the platelets were sufficient in number and were primarily organized in clumps (Figures 3, 4).

**Figure 5:**
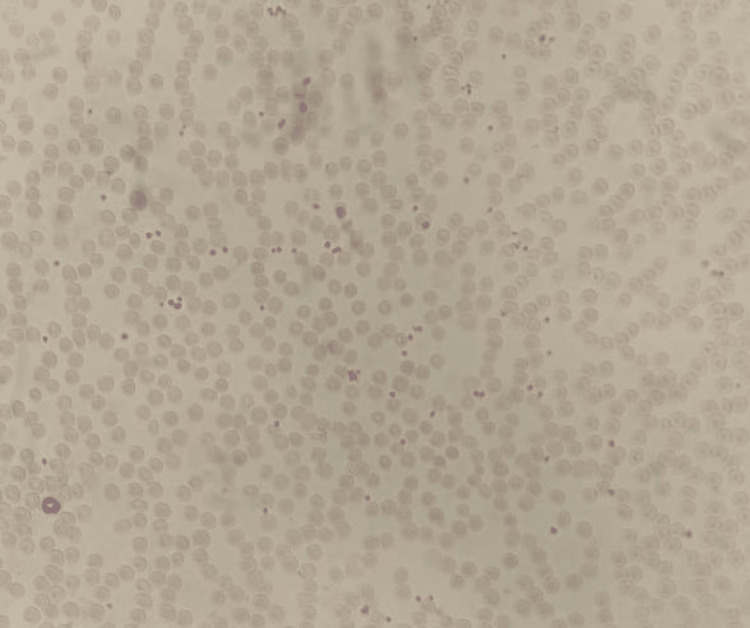
A peripheral smear performed using sodium citrate as the anticoagulant showed no platelet clumping and a normal platelet count

Consequently, the analysis was repeated with sodium citrate as the anticoagulant, which revealed a normal platelet distribution and no platelet clumping (Figure 5).

**Figure 6:**
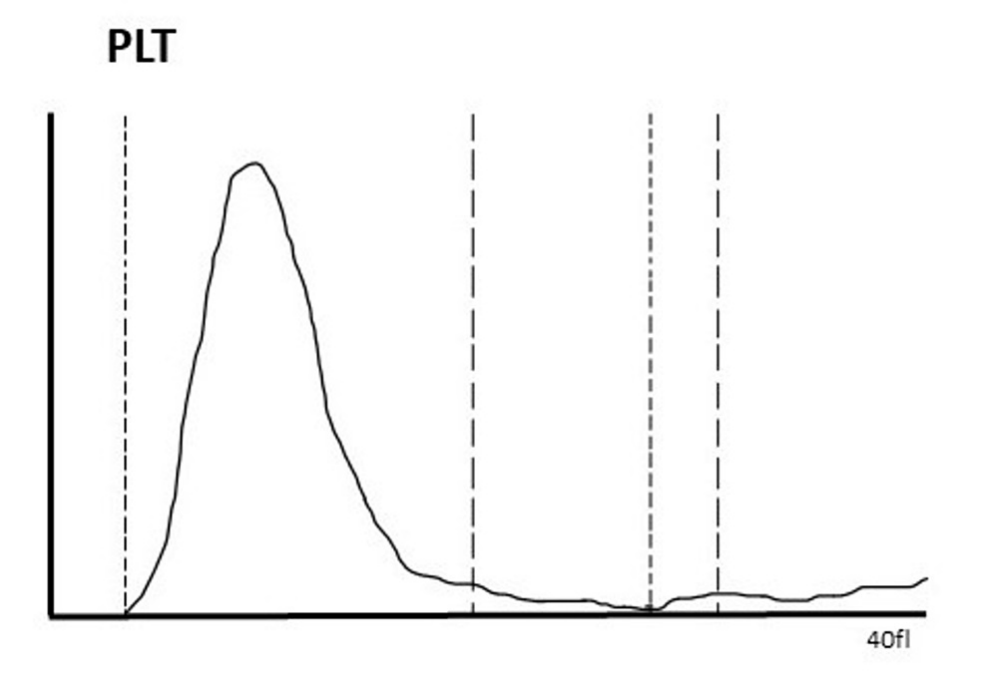
A histogram of the sample anticoagulated with sodium citrate shows a platelet count of 202,000 cells/mm3. PLT: platelet

The platelet count was 202,000/mm3 when it was determined using sodium citrate as the anticoagulant on an automated analyzer, with a platelet histogram showing a normal smooth curve (Figure 6).

Considering the absence of any clinical symptoms in the patient, the presence of platelet clumps coupled with low platelet counts when EDTA was used as the anticoagulant, and the normalization of platelet counts when citrate was used as the anticoagulant, a diagnosis of EDTA-induced pseudo thrombocytopenia was made. The patient was made aware of her diagnosis and was also informed to let any further investigating physician know that she has EDTA-PTCP, which can decrease unwanted excessive evaluation.

## Discussion

EDTA-induced pseudo thrombocytopenia (EDTA-PTCP) is a laboratory phenomenon that is characterized by platelet clumping, which can be seen on a peripheral smear, and an abnormal platelet histogram with a typical sawtooth pattern. The incidence is about 0.1-0.2% in the general population [1-3], which makes it an important differential for thrombocytopenia without any pathological causes. It is more commonly seen in women under the age of 50, and there is a slight male predominance in age groups over 50 [5]. Studies have shown that this phenomenon is not necessarily associated with any disease, and the patients do not show any clinical features of thrombocytopenia, but it can rarely be a serendipitous finding in association with a disease or syndrome [5]. It is important for physicians to have a high degree of suspicion about this phenomenon, especially when dealing with asymptomatic patients showing thrombocytopenia on repeated tests.

A frequently asked question is the possibility of transmission of PTCP to the recipient after blood transfusion, plateletpheresis, or peripheral blood stem cell transplant. As per the available data and case reports, it has been repeatedly concluded that PTCP should not be a reason to withhold any of the aforementioned procedures and is usually not transferred to the recipient [11-14]. However, theoretically, there is a chance of developing PTCP in peripheral blood stem cell transplantation, especially after the development of the B-cell repertoire, which might take a significant amount of time and was observed in a case reported by Di Francesco et al. [15]. Hence, it is also important to examine the patient’s peripheral smear after a certain period following the stem cell transplant, which will allow mature B-cells to repopulate the recipient’s bloodstream. Neonates can also display pseudo thrombocytopenia due to the transplacental transmission of maternal EDTA-dependent antibodies [16, 17].

Scrub typhus was found to be associated with PTCP, which was transient for a few weeks after treatment [18,19]. This is an important association because scrub typhus can also be associated with true thrombocytopenia, which might require platelet transfusions. Hence, one has to be vigilant in such a pathological setting. Abciximab, an anticoagulant that acts by preventing fibrinogen and von Willebrand factor from binding to the glycoprotein (GP) IIb/IIIa receptor on platelets, has also been associated with PTCP [20]. There was a significant association noted between PTCP and viral infections, especially hepatitis A, followed by cytomegalovirus and H1N1 influenza (swine flu) [21]. Apart from the abovementioned scenarios, PTCP is also observed in patients with bladder cancer [22], autoimmune thrombocytopenia [23], SARS CoV2 infection, post-Oxford-AstraZeneca COVID-19 vaccine [24, 25], and Graves’ disease [26].

**Figure 7:**
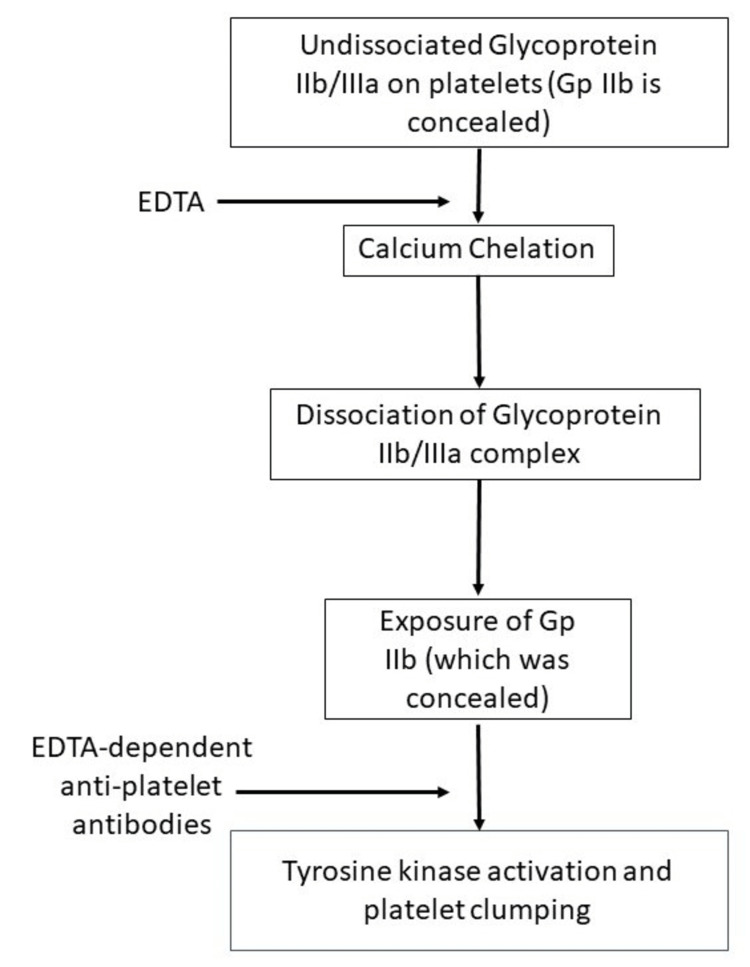
The process of formation of platelet clumps causing pseudo thrombocytopenia [27,28] EDTA: ethylene diamine tetra acetate Image Credits: Adarsh Vardhan Tangella (Author)

EDTA-PTCP is primarily precipitated by the presence of EDTA-dependent anti-platelet antibodies (27). The following flowchart (Figure 7) summarizes the pathophysiology of this phenomenon (27, 28).

IgG and IgM-class antibodies are most frequently produced in response to the abovementioned phenomenon, although IgA antibodies can also be found and can lead to cold agglutination [5]. Among the IgG subtypes, IgG1 is seen most frequently [4]. It is to be noted that there is a progressive thrombocyte count decline as the duration between sample collection and testing increases due to platelet clumping, and the thrombocyte count is more stable at 4^o^C compared with other temperatures. This shows that the period between sample collection and testing is an important parameter to consider when trying to alleviate PTCP. Hence, it is important to measure platelet counts as early as possible after sample collection [29].

Peripheral smear examination is the gold standard to identify platelet clumping and PTCP, which was evident in our case report [1]. Another typical finding of the automatic analyzer report is a sawtooth pattern of platelet histogram with an abnormal height, which was also observed on the histogram presented previously in our case report [1]. This is an easier way to identify this spurious laboratory phenomenon, as it can become difficult to perform peripheral smears in every case. Proper awareness about platelet clumping, the use of smear examination, and a high degree of suspicion can guide physicians in preventing unnecessary treatment in patients with PTCP. It should be noted that PTCP is not limited to EDTA. It can also be seen with heparin, oxalate, hirudin [30,31], and, in some cases, citrate [5]. The following step-by-step evaluation of blood samples (as described by Waseem et al.) can help in identifying the anticoagulants causing PTCP [32]:

Step 1: A blood sample is collected in EDTA. If clumping persists, continue to step 2. Step 2: The blood sample is collected in sodium citrate. If step 2 is not possible, proceed to step 3. Step 3: Obtain a sample in lithium heparin. If clumping is still present, then perform step 4. Step 4: Obtain a sample in sodium fluoride. If clumping is still present, then perform step 5. Step 5: Obtain a sample in ammonium oxalate and count platelets using a Neubauer chamber, if available. Steps 3, 4, and 5 are usually needed in rare circumstances where steps 1 and 2 do not solve the issue of platelet clumping.

Vortex-mixing of samples with platelet clumps has also shown a reduction in platelet clumping and platelet satellitism without any changes in RBC, WBC, or schistocyte counts [33].

Magnesium sulfate has been known to be a decent replacement for EDTA as an anticoagulant in cases of EDTA PTCP [27,28]. Magnesium sulfate does not cause GP IIb/IIIa dissociation by preventing the exposure of the hidden GP IIb component and maintaining the integrity of the complex. This prevents the binding of EDTA-dependent antiplatelet antibodies to the gp IIb subunit, resulting in a reduction in platelet clump formation [34]. It has also been noted that prewarming blood samples to 37^o^C and adding kanamycin to the sample reduces platelet clumping and PTCP [35,36]. Other aminoglycosides might also have similar effects on PTCP [5,36]. A novel method of platelet clump dissociation, which is as efficacious as kanamycin, has been devised using a combination of 9 mmol/L CaCl2 and 0.1 unit/L sodium heparin [37]. Apart from these, a mixture of citrate, pyridoxal-5-phosphate, and Tris (CPT) can also be used to prevent PTCP [5].

## Conclusions

It is crucial to double-check lab results obtained with automated analyzers in each patient with thrombocytopenia (especially when it is unrelated to any clinical features) by examining the peripheral smear in order to find any platelet aggregates that can help confirm EDTA-PTCP and avoid unnecessary investigatory procedures.
